# Introduction to 2D materials and their applications

**DOI:** 10.1039/d4ra90059f

**Published:** 2024-06-03

**Authors:** Diana Berman, Agnieszka Jastrzebska, Massimiliano Papi, Andreas Rosenkranz

**Affiliations:** a Department of Materials Science and Engineering, University of North Texas Denton USA diana.berman@unt.edu; b Division of Microtechnology and Nanotechnology, Institute of Metrology and Biomedical Engineering, Faculty of Mechatronics, Warsaw University of Technology św. A. Boboli 8 02-525 Warsaw Poland agnieszka.jastrzebska@pw.edu.pl; c Dipartimento di Neuroscienze, Università Cattolica del Sacro Cuore Largo Francesco Vito 1 Rome 00168 Italy massimiliano.papi@unicatt.it; d Fondazione Policlinico Universitario A. Gemelli IRCSS Rome 00168 Italy; e Department of Chemical Engineering, Biotechnology and Materials (FCFM), Universidad de Chile Santiago Chile arosenkranz@ing.uchile.cl

## Abstract

Diana Berman, Agnieszka Jastrzebska, Massimiliano Papi, and Andreas Rosenkranz introduce the *RSC Advances* themed issue on 2D materials and their applications.
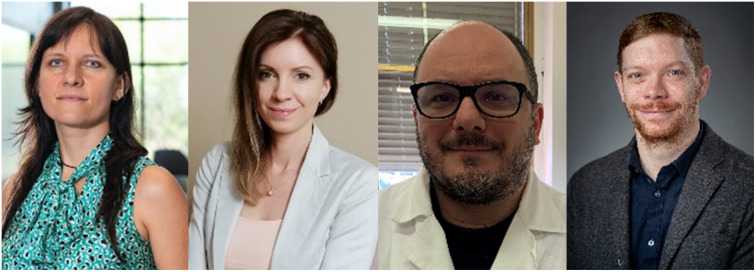

In recent decades, 2D materials have gained notable attention due to their extraordinary structural and physicochemical attributes. These materials, characterized by their two-dimensional arrangement of atoms, have emerged as versatile tools with applications spanning a multitude of disciplines, captivating researchers worldwide.^1^

This fascination can be traced back to the groundbreaking synthesis of graphene in 2004, a pivotal moment that sparked a surge of interest and launched investigations into novel 2D materials.^2–5^ Inspired by graphene's performance, scientists uncovered, designed, and engineered a diverse array of 2D nanomaterials, each harbouring unique properties and promising functionalities. Advanced synthesis methodologies have been developed, enabling the realization of materials such as silicene, hexagonal boron nitride, black phosphorus, metal–organic frameworks, covalent-organic frameworks, MXenes, and MBenes.^6–9^

Researchers have investigated the fundamental mechanisms governing the behaviour of 2D materials, with an emphasis on the intricate relationships between structure, property, and performance. 2D materials have emerged as frontrunners in a range of applications, due to their high surface-to-volume ratios, good mechanical properties, controllable electrical and thermal conductivities, and chemical tunability. From revolutionizing energy storage and harvesting technologies to catalysing advancements in (photo)-catalysis and water purification, 2D materials impact across an array of fields.^6–8,10,11^

Moreover, the allure of 2D nanomaterials extends beyond traditional domains, encompassing emerging frontiers such as wearable electronics, biomedicine, and tribology.^7,8,12^ With each passing discovery, the boundaries of possibility expand, offering glimpses into a future where these materials play a pivotal role in addressing pressing societal challenges and driving technological innovation.

As we follow this journey of exploration and innovation, the landscape of materials science undergoes a profound transformation. Therefore, this themed collection is expected to serve as an innovative platform to share the latest, cutting-edge research trends and findings related to 2D materials. From a more fundamental point of view, Uddin *et al.* (https://doi.org/10.1039/D3RA04456D) summarize the recent progress and challenges of graphene-like materials, including transition metal dichalcogenides, boron nitride and MXenes. Their review serves as an excellent guide for application-related research beyond graphene.

Regarding graphene-based nanocomposites, Shin *et al.* (https://doi.org/10.1039/D3RA08946K) assess and discuss their performance in biomedical applications, including biosensing, drug delivery, and tissue engineering. In terms of graphene-related original articles, Kwon *et al.* (https://doi.org/10.1039/D3RA08932K) present an innovative study on the interfacial design of mechanically robust nanocomposite films. Based on graphene oxide, these films exhibit enhanced tensile strength, modulus, and toughness. Further, the incorporation of Ag nanoparticles induced catalytic activity thus creating multi-functional composite films. Alsulami *et al.* (https://doi.org/10.1039/D3RA03748G) successfully fabricate graphene-nanodiamond composite films using microwave-assisted synthesis to study their thermoelectric properties. These thermoelectric properties are shown to be dependent on temperature and material properties. Kurus *et al.* (https://doi.org/10.1039/D3RA07018B) conduct a fundamental study on surface and tip-enhanced Raman spectroscopy of graphene. The authors confirm that tip-enhanced Raman spectroscopy in gap mode is capable of sensing and detecting structural and topographical variations as well as changes in the mechanical state of monolayer graphene, with nanoscale resolution. They also confirmed a local plasmonic enhancement with a factor of 100 using surface-enhanced Raman spectroscopy. This study innovatively paves the way for advanced nanoscale characterization of structural features in graphene, which may be soon extrapolated to other 2D materials. Pradeepkumar *et al.* (https://doi.org/10.1039/D3RA08289J) investigates the possibility of epitaxially growing graphene on SiC surfaces for photonic and electronic devices using Ni/Cu as a catalyst at elevated temperatures.

Beyond graphene-based materials, several interesting contributions have been published related to other 2D materials used in various applications. In this context, Bobbitt *et al.* (https://doi.org/10.1039/D3RA07984H) computationally using Monte Carlo simulations, assess the adsorption behaviour of water on MoS_2_ surfaces and edges. Adsorption probability was greatly influenced by the existing defect situation, implying that defective surfaces became saturated at low relative humidities. Moreover, they demonstrated that water tends to bind to surface defects, and once the defects are saturated, MoS_2_ edges become the preferred adsorption sites. Garnes-Portolés *et al.* (https://doi.org/10.1039/D3RA07331A) study few-layer black phosphorous regarding its nitrogen fixation potential. They demonstrate that delaminated black phosphorous tends to oxidize under ambient conditions, which helps to fix/adsorb nitrogen on the modified surfaces. This study sheds light on the fundamental chemical processes and environmental stability of black phosphorous, which is essential for many applications. Karuppaiah *et al.* (https://doi.org/10.1039/D3RA05629E) develop an elective sensor for kojic acid (KA), which is frequently used in cosmetics, pharmaceuticals, and food items. The sensor was based on a glassy carbon electrode with a nanocomposite consisting of Ti_3_C_2_, and Ag nanoparticles, which increased sensitivity and reduced the resulting impedance, optimizing sensor performance. Sun *et al.* (https://doi.org/10.1039/D3RA03669C) designed and synthesized dual-emissive fluorescent silicon quantum dots for cell imaging applications. The fabricated quantum dots induced stable, dual-emissive fluorescence emissions observed in the visible and near-infrared range, with both peaks suitable for bioimaging purposes. The results clearly demonstrated that the designed approach was non-toxic and applicable to a variety of cell types.

We, the guest editors, feel very proud of the results published in this themed collection. Through collaborative efforts and interdisciplinary collaboration, the potential of 2D materials unfolds, offering opportunities limited only by the bounds of imagination. Across theoretical and experimental studies, this collection covers various 2D materials and different application types.

We extend our appreciation to the authors who have contributed to this collection. Their efforts, coupled with the discerning insights of our referees, have collectively shaped a rich collection. Yet, as we delve into the realms of 2D materials, we are reminded of the relentless march of progress. It is our hope that this collection serves not merely as a static repository of knowledge, but as a catalyst for further inquiry and exploration.

As we navigate the ever-expanding frontier of materials research, we invite our readers to join us on this journey of discovery. From graphene's remarkable conductivity to the tantalizing properties of transition metal dichalcogenides, the possibilities of 2D materials are as boundless as the imagination itself. Together, let us keep a keen eye out for the latest trends in this exciting field.

## Supplementary Material
